# Genomic and Bioinformatics Approaches for Analysis of Genes Associated With Cancer Risks Following Exposure to Tobacco Smoking

**DOI:** 10.3389/fpubh.2018.00084

**Published:** 2018-03-20

**Authors:** Mohammed A. I. Al-Obaide, Buthainah A. Ibrahim, Saif Al-Humaish, Abdel-Salam G. Abdel-Salam

**Affiliations:** ^1^Department of Biomedical Science, School of Pharmacy, Texas Tech University Health Science Center, Amarillo, TX, United States; ^2^Department of Physics, Diyala University, Baquba, Iraq; ^3^Biomedica, LLC, Sterling Heights, MI, United States; ^4^Department of Mathematics, Statistics and Physics, College of Arts and Sciences, Qatar University, Doha, Qatar

**Keywords:** tobacco smoking, bioinformatics, cancer, single-nucleotide polymorphisms, DNA methylation, carcinogenic chemicals, health risk

## Abstract

Cancer is a significant health problem in the Middle East and global population. It is well established that there is a direct link between tobacco smoking and cancer, which will continue to pose a significant threat to human health. The impact of long-term exposure to tobacco smoke on the risk of cancer encouraged the study of biomarkers for vulnerable individuals to tobacco smoking, especially children, who are more susceptible than adults to the action of environmental carcinogens. The carcinogens in tobacco smoke condensate induce DNA damage and play a significant role in determining the health and well-being of smokers, non-smoker, and primarily children. Cancer is a result of genomic and epigenomic malfunctions that lead to an initial premalignant condition. Although premalignancy genetic cascade is a much-delayed process, it will end with adverse health consequences. In addition to the DNA damage and mutations, tobacco smoke can cause changes in the DNA methylation and gene expression associated with cancer. The genetic events hint on the possible use of genomic–epigenomic changes in genes related to cancer, in predicting cancer risks associated with exposure to tobacco smoking. Bioinformatics provides indispensable tools to identify the cascade of expressed genes in active smokers and non-smokers and could assist the development of a framework to manage this cascade of events linked with the evolvement of disease including cancer. The aim of this mini review is to cognize the essential genomic processes and health risks associated with tobacco smoking and the implications of bioinformatics in cancer prediction, prevention, and intervention.

## Introduction

Direct involvement of chemicals in the tobacco smoke with cancer has been shown since the 1970s and considered as pleiotropic carcinogens that cause DNA damage (1–3). Although, tobacco smoke associated with many types of cancer, it is linked primarily to lung cancer in 90% of men and 70–80% of lung cancer in women (4). Furthermore, tobacco smoke causes more than 13 types of cancer in men and women (5–11). The main consequences of long-term exposure to tobacco smoke associated with the premalignancy mechanism, which is a considerably delayed process, sometimes taking several years (12, 13). Accordingly, cancer surveillance plays an essential role in cancer prevention and intervention of various socioeconomic aspects associated with cancer attributed to tobacco smoking. The health risk of tobacco smoke is of utmost importance to assess in children and non-smokers. These health issues require sophisticated genomic assays besides the clinical procedures (14–16). The incidence of disease and cancer risks from cigarette smoking among male and female smokers, increased in the Middle East and worldwide over most of the twentieth century, and continued in the twenty-first century as compared with persons who have never smoked (5, 6, 17–19).

In this study, we will highlight genomic and molecular features, which can be used to develop approaches to predict cancer after long-term tobacco smoke exposure. Most importantly, the influence of tobacco smoke on children and non-smokers. Unfortunately, the negative impact of tobacco smoke on the health of non-smokers is a result of involuntary exposure to tobacco smoke. It is, therefore, crucial that the outcome of research efforts in this field go hand in hand with efforts to develop practical informatics and bioinformatics programs to face the challenges through good practices and to strengthen public awareness and understanding of hidden dangers of tobacco smoking. Thus, research activities in this area will participate in creating more efficient cancer prevention program and find novel molecular methods for early detection of premalignancy lesions caused by tobacco smoke carcinogens.

## Carcinogenic Chemicals in Tobacco Smoke

**Figure 1:**
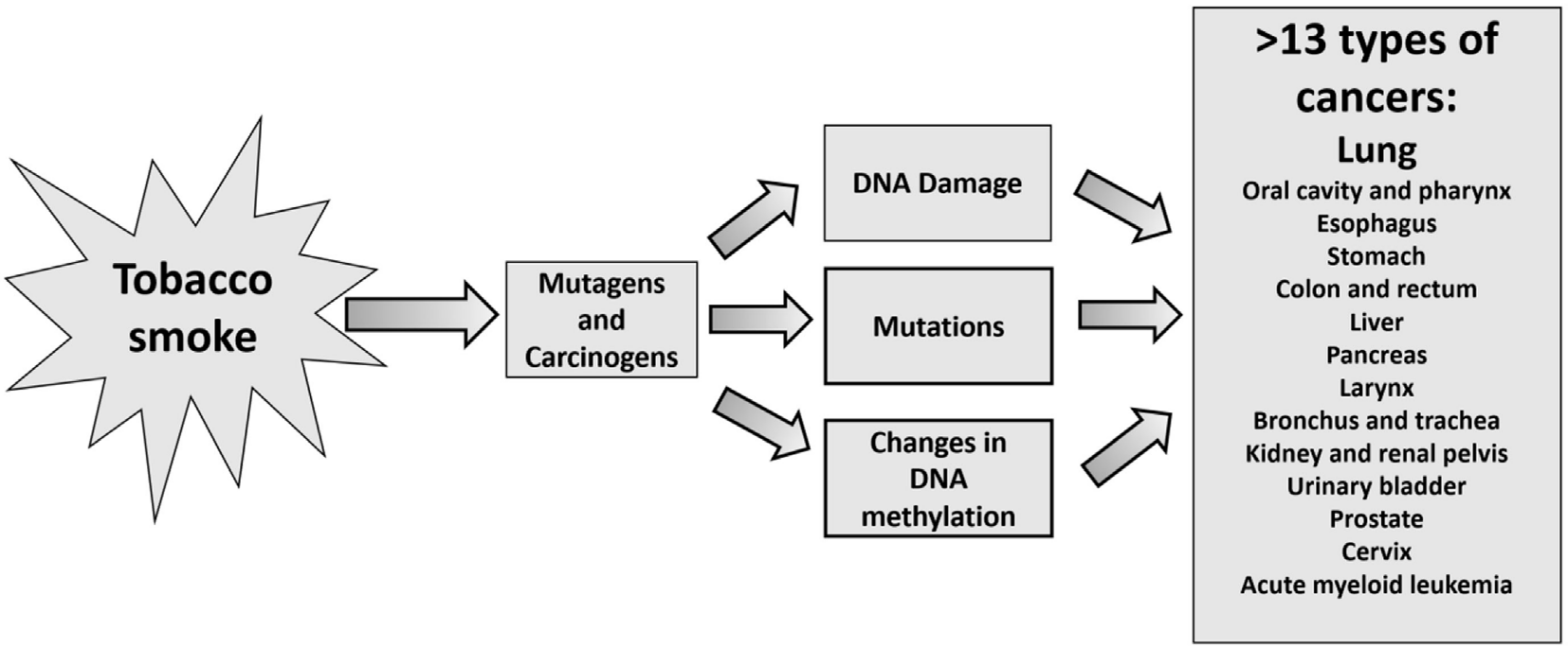
Events associated with tobacco smoke leading to more than 13 types of cancers.

Tobacco smoke constitutes more than 4,000 compounds, of which over 70 with carcinogenic activities, such as polycyclic aromatic hydrocarbons (PAHs), nitrosamines, aromatic amines, and others (6, 20, 21). The most critical nitrosamines carcinogens in tobacco smoke are *N*-dimethylnitrosamine (DMN, also known as *N*-nitrosodimethylamine), 4-(methylnitrosamino)-1-(3-pyridyl)-1-butanone (NNK), and *N*′-nitrosonornicotine (NNN) (1–3). *N*-nitrosamines and tobacco-specific nitrosamines (TSNAs) can attack DNA at the O6-position of guanine forming DNA adducts O6-methylguanine and O(6)-[4-oxo-4-(3-pyridyl) butyl]guanine (O6pobG). Both are mutagenic lesions (22), if left unrepaired would pair with thymine and introduce G:C to A:T transition upon DNA replication. Another study showed no reduction observed in tobacco carcinogenic nitrosamines associated with cancer (23). The study revealed that the sum of the two potent carcinogens, NNK and NNN in cigarette filler averaged 2.54 ± 1.05 μg/g tobacco, which is identical to the results of these two carcinogens reported for the tobacco of a U.S. filtered cigarette in 1979. Tobacco smoke carcinogenic chemicals can cause DNA damage and can give rise to mutations and single-nucleotide polymorphisms (SNPs) in genes and regulatory sequences associated with various types of cancers. DNA damage caused by tobacco smoke carcinogens is related to cancer development (2) and found associated with lung cancer (4). Interestingly, though tobacco use causes more than 13 types of cancer in men and women (5–11), it is the leading preventable cause of cancer (6). Figure 1 summarizes the events followed exposure to tobacco smoke and development of cancer.

## Consequences of Environmental Tobacco Smoke (ETS)

The health risks because of inhaling tobacco smoke are not limited to tobacco smokers. Because cancer risks owing to tobacco smoke inhalation can extend to non-smokers especially children, who involuntarily inhale ETS at home, at work, or in public places (24). More recent studies showed the non-smokers represent a significant percentage of lung cancer patients, which is estimated approximately 25% of all lung cancer cases indicating the deadly effect of passive and second-hand smoking (25). Further, children and non-smokers exposed to third-hand smoke (THS), which is residual tobacco smoke deposited on surfaces and dust and linked to higher cancer risk incidence in non-smokers (26). The study showed the likely cancer risk by age group *via* dermal exposure to carcinogen *N*-nitrosamines and TSNAs measured in house dust samples. The calculated TSNAs risks surpassed the upper-bound risk advocated by the United States Environmental Protection Agency (EPA/U.S. EPA).

The consequences of risks following exposure of children and non-smoker, the main victims of the ETS and THS, to TSNAs are not limited to the ability of TSNAs to induce DNA damage and play a significant role in determining the health and well-being of children. But extended to the association of mutagenic, carcinogenic and DNA-damaging effects of various chemicals in tobacco smoke inhalation associated with the premalignancy mechanism (12, 13), which is a much-delayed process, sometimes taking as long as 30 years. Although there is some controversial association between parental smoking during pregnancy and risk of childhood tumors (27), recent studies have reported positive associations between paternal smoking during pregnancy and childhood brain tumor risk (28, 29) and childhood acute lymphoblastic and myeloid leukemia (30).

## Tobacco Smoke, SNPs, and Cancer

Mutations are considered the primary sources of SNPs in genes leading to the formation of genetic variants linked to the development of cancer following exposure to environmental risk factors (31–33). Many SNPs located within the regulatory regions of the genes, which may influence the expression of the genes (34, 35). SNPs of glutathione S-transferase (*GST*) and *TP53* are examples. The GST enzymes contribute to the activation and inactivation of oxidative metabolites of carcinogenic agents correlated with cancer (36). The identified polymorphisms in GST genes associated with cancer susceptibility and increased the risk resulting from exposure to tobacco smoke and decreased the ability of GST enzymes to detoxify the carcinogens (28, 36). The TP53 gene is a tumor suppressor gene and involved in several vital cellular functions (37, 38). The *TP53* gene harbors high frequency of functional SNPs, which may alter P53 protein function (39). Studies reported the association of several TP53 SNPs with risk of cancer. Mutations in the *TP53* tumor suppressor gene implicated in different cancer types, including lung cancer (40–42). Multifactor dimensionality reduction analysis used and provided evidence on interactions among various *P53* SNPs and tobacco habit (43).

## Impact of Tobacco Smoke on Genomic DNA Methylation

DNA methylation plays a significant role in silencing gene expression, and changes in genomic DNA methylation patterns from the natural state will lead to the development of cancers (44, 45) (Figure 1). Tobacco smoke causes abnormal DNA methylation profiles in the genome of smokers and involuntarily exposed non-smokers. Evidence of significant differences observed in the degree of site-specific methylation in each of the 22 autosomes as a function of tobacco smoking (46), suggesting DNA methylation aberration is another criterion associated with the prediction of cancer risk. The epigenetic changes observed at CpGs in blood. Su et al. (47) identified novel CpGs associated with current smoking, pack-years, duration. The authors were able to show unique profiles of smoking-associated DNA methylation and gene expression among immune cell types. Another study showed from CpG site methylation that tobacco smoking differently influences cell types of the innate and adaptive immune system-indications (48). The link between lung cancer-related genes and tobacco smoking was confirmed through identifications of 13 novel CpG with a decrease of DNA methylation compared to never smoking sites located in 8 genes: *KLF6, STK32A, TERT, MSH5, ACTA2, GATA3, VTI1A*, and *CHRNA5* (49).

## Bioinformatics Applications in Tobacco Smoke-Associated Cancer Genes

There are several definitions of “Bioinformatics,” in this article we use NIH working definition of bioinformatics “Research, development, or application of computational tools and approaches for expanding the use of biological, medical, behavioral or health data, including those to acquire, store, organize, archive, analyze, or visualize such data.” The U.S. National Library of Medicine gives a similar definition “The organization and analysis of biological and related information, usually involving the use of computers to develop databases, retrieval mechanisms, and data analysis tools, especially in the fields of molecular biology, structural biology, and genetics.” Bioinformatics applies principles of information sciences and technologies to make the vast, diverse, and complex life sciences data more understandable and accessible. Thus, it is the bridge between domain users (i.e., researchers) to the software needed to support the workflow of their domain. Here, we show an example that used bioinformatics to develop a knowledgebase for addiction-related genes (KARG) database (50). KARG is a cross-platform database links genes and chromosome regions and pathways to addiction. An important accomplishment of the database identification of pathway for addiction chemicals. The KARG database identified addiction pathway of nicotine, which is a result of the neuroactive ligand-receptor interaction.

Furthermore, bioinformatics databases can provide indispensable tools to assist identification of changes in the expression of genes associated with nicotine addiction in active smokers and never smokers (see Discussion). Also, the bioinformatics databases can provide a framework for scientists to manage the deluge of data and could help to discover new genes associated with tobacco smoking addiction and initiation. The data from NCBI-Gene and UniProt were used to show the expression and health risks of tobacco smoking associated with nine genes related to the initiation of smoking and nicotine dependence, and four genes linked to lung cancer (Table 1). The table also shows the potential to discover a new gene, *KMO* that can help in tobacco smoking cessation. Modulating *KMO* gene expression could be a useful tactic for the treatment of tobacco dependence (51). Collectively, the data show the bioinformatic methods help to investigate the genes’ involvement in tobacco smoking initiation and dependence, cancer and other diseases and therapy.

**Table 1:** Examples of genes associated with health risks of tobacco smoking.

Gene	Protein	Smoking trait	Disease	Gene expression	Reference
*CHRNA3*	Neuronal acetylcholine receptor subunit alpha-3	Initiation, dependence	Lung cancer, obstructive pulmonary	0.197 ± 0.088^a^	(52–55)
*CHRNA5*	Neuronal acetylcholine receptor subunit alpha-5	Initiation	Obstructive pulmonary, lung and colorectal cancer	0.234 ± 0.194^a^	(52, 55–57)
*CHRNB3*	Neuronal acetylcholine receptor subunit beta-3	Dependence	Esophageal adenocarcinoma	0.132 ± 0.071^a^	(55, 58, 59)
*CHRNB4*	Neuronal acetylcholine receptor subunit beta-4	Dependence	Lung cancer	0.022 ± 0.016^a^	(55, 60)
*CYP2A6*	Cytochrome P450 2A6	Dependence	Lung cancer	542 ± 278^b^	(55, 61–63)
*NTRK2*	BDNF/NT-3 growth factors receptor	Nicotine dependence	Mood disorders	64.619 ± 20.74^a^	(52, 64)
*GABARAP*	Gamma-aminobutyric acid receptor-associated protein	Nicotine dependence	–	148.01 ± 24.08^a^	(52, 65)
*ANKK1*	Ankyrin repeat and protein kinase domain-containing protein 1	Nicotine dependence	Attention deficit hyperactivity disorder	0.01 ± 0.01^a^	(52, 66)
*KMO*	Kynurenine 3-monooxygenase	Addiction therapy	Huntington disease, schizophrenia	1.976 ± 0.559^a^	(51, 52)

*The data extracted from NCBI-Genes and proteins, and UniProt databases. The measurements of expression of genes presented as mean Reads Per Kilobase Million in the ^a^brain and ^b^liver*.

## Discussion

The bioinformatics methods used in the analyses of genomic sequences and gene expression profiles of genes and regulatory elements can identify the changes that arise because of exposures to tobacco smoke. The induced changes in tobacco smoke cause mutations and form SNPs, and malfunction of regulatory modes of gene expressions (36, 49). Many chemicals and carcinogens identified in tobacco smoke are linked to several human malignancies and other diseases (Table 1). Tobacco smoking can cause cancer, because of *N*-nitrosamines and TSNAs (22, 23), recent studies showed that nicotine is associated with the development of cancer (67). This finding exacerbates the health risks of nicotine addiction and suggests its involvement in worsening and relapse of cancer, considering the formation of TSNAs from nicotine in the body (67–69).

The data from NCBI-Gene and Uniprot showed the link between tobacco smoking and health risk of eight genes (Table 1). Although *GABARAP* showed no health risk, its involvement in nicotine addiction suggested the potential formation of TSNAs from nicotine and development of cancer (67–69). The table also shows the potential to discover the new gene *KMO* that can help in tobacco smoking cessation. Modulating *KMO* gene expression could be a useful tactic for the treatment of tobacco dependence (50). The expression *KMO* is reduced in schizophrenia and in bipolar patients with a history of psychosis (50), and two *KMO* SNPs were found frequently in schizophrenia patients compared with healthy controls (51).

Integrated bioinformatics approaches were used to show the differentially expressed genes (DEGs) in smokers and non-smokers. Cao et al. (70) used in their analysis of DEGs: gene ontology, pathway enrichment analyses of DEGs, protein–protein interaction network, and transcriptional regulatory network along with miRNA-target regulatory network construction. The authors showed DEGs, for example, *CYP1A1, CYP1B1, YWHAZ*, and *PTPRD*, may have the potential to be used as biomarkers and therapeutic targets in tobacco smoke-related pathological changes. Bioinformatic methods also showed applications in investigating the impact of tobacco smoke on regulatory factors associated with gene expression in various ways, for example, an aberration in DNA methylation (46–49, 71, 72), transcriptional binding sites (73), and noncoding RNA (70, 74, 75).

Another topic requires consideration is the correlation between medical informatics and bioinformatics. In their article, Wyatt and Liu suggested that medical informatics include bioinformatics, clinical informatics, consumer health informatics (CHI), and public health informatics (76). Bio and medical informatics have applications in various fields specifically, genomic medicine. Generally, their contributions were in areas such as knowledge-based systems, database design, data mining, genomic sequences, and structure analysis (77). Of interest, CHI, which is interacting topic with bioinformatics. In their report, Gibbons et al. (78) showed the outcomes related to the use of tobacco that included self-management, program adherence, and change in health behaviors. The outcome indicates that CHI applications had statistically significant effects on health.

Finally, the main enigma of health risk associated with tobacco smoking is not restricted to cancer but it is also linked to other noncommunicable diseases (NCDs), cardiovascular diseases, chronic respiratory, and diabetes (79, 80). Tobacco smokers with diabetes have higher risks for serious cardiovascular complications (81). The NCDs, are the major reason for mortality in the world that represent 63% of all annual deaths, over 36 million people each year. Intriguingly, nearly 80% of all NCDs deaths take place in low and middle-income countries (80). The impact of the sociodemographic and psychosocial factors associated with men and women is apparent in emerging adulthood who are prone to the tobacco smoking habit. Nowadays, although smoking prevalence and cigarette consumption have declined in some countries, smoking prevalence among women is increasing (20, 82–87).

In conclusion, tobacco smoke is a widespread carcinogen; which contains many chemicals known to cause cancer, including PAHs, nitrosamines, and aromatic amines. It can cause DNA damage, mutations, and changes in DNA methylation. A considerable percentage of lung cancer is attributable to ETS carcinogens, besides more than 13 other types of cancer and NCDs. Applying bioinformatics, medical informatics and genomic for cancer surveillance in smokers and non-smokers could be utilized in cancer prediction, prevention, and intervention.

## Author Contributions

The authors have made equal contributions in the writing and revising of this mini review.

## Conflict of Interest Statement

The authors declare that the research was conducted in the absence of any commercial or financial relationships that could be construed as a potential conflict of interest.
